# Message from the Editor-in-Chief

**DOI:** 10.3390/life5010212

**Published:** 2015-01-13

**Authors:** Pabulo Henrique Rampelotto

**Affiliations:** Center of Biotechnology and PPGBCM, Federal University of Rio Grande do Sul, Bento Gonçalves Avenue, P.O. Box 15005, 91501-970, Porto Alegre—RS, Brazil; E-Mail: p.rampelotto@mail.ufsm.br

One year after I assumed the position of Editor in Chief of *Life*, it is my great pleasure to write this editorial highlighting our achievements during this period, which were so many!

As I wrote in the first editorial, my commitment was to make the journal a success, with the launch of exciting special issues, publication of high quality papers as well as inclusion of the journal in major indexing and abstracting services [1]. Basically, throughout 2014, all these commitments have been accomplished. Several eminent team leaders have joined our editorial board [2], timely special issues have been launched [3], and the journal already was indexed in PubMed [4]; now, it is just a matter of time to be indexed in Web of Science. The feedback from the scientific community already has been quite positive. With timely special issues lined up for 2015, the journal is developing quite fast.

In my inaugural editorial as Editor in Chief, I highlighted that *Life* would provide a forum for the publication of new hypotheses aiming to encourage discussion and creative hypothesis testing by members of the scientific community. Now, the journal has a specific section dedicated to this important theme, *i.e.*, *Hypotheses in the Life Sciences* [5].

In collaboration with MDPI, I have implemented a new system of open peer review in order to demonstrate the rigorous, fair and efficient standard of our editorial work [6]. The first paper published under this new policy was a manuscript written by a Nobelist and reviewed by three experts in the field [7]. I am pleased to demonstrate that papers from authors of such high standing undergo the same rigorous process as any other.

For 2015, I am also preparing some new interesting features, such as publishing the feedback from the authors who have published in our journal. This transparency is very important because there is little information in the literature about the quality of the peer review process and the time it takes. So far, this kind of information, which is usually hidden by the journals, passes between colleagues; you might hear that some journals are particularly slow in processing, whereas others are known for their effectiveness. However, when you try to find something more concrete on the matter, the information is rather fragmented and incomprehensive. In order to provide systematic and clear information to the scientific community, I am working to make the editorial work in *Life* more transparent by sharing the author’s testimonials about the entire editorial process, its quality, and the time it has taken. This will highlight the quality of our work by the words of those who have worked closely with us. At a time when the review process in scientific journals can take several months, and an additional several months until the paper is officially published, a platform for fast, efficient and cost-effective publication without undue delay or expense is essential for any scientific research.

By the achievements pointed out in this editorial, which covers only one year (!), you may have an idea of how committed we are in providing a high quality journal to the scientific community, with all the advantages of an open access platform. I truly believe that the open access movement provides the setting to radically reform scientific publishing, and I am making every effort to accomplish that with *Life*. Hope you have the pleasure of working with us in the future. Your manuscript and feedback will be most welcome!
